# Flexor Carpi Radialis and Dorsal Intercarpal Complex Elongation After 3 Ligament Tenodesis

**DOI:** 10.1177/15589447251404957

**Published:** 2025-12-29

**Authors:** Daniel Bakker, Steven Merkens, Niek Parser, Joost Colaris, Gert-Jan Kleinrensink, Nina Mathijssen, Gerald Kraan

**Affiliations:** 1 Reinier de Graaf Groep, Delft, The Netherlands; 2 Erasmus Medical Center, Rotterdam, The Netherlands

**Keywords:** biomechanics, capsulodesis, kinematics, scapholunate interosseous ligament (SLIL), SL-ligament, SL-interval

## Abstract

**Background:**

To improve the surgical procedure and rehabilitation after surgery, a better understanding of the biomechanical properties of the 3 ligament tenodesis (3LT) procedure is necessary.

**Method:**

This study examined elongation in relation to flexion, and type of failure after 3LT using 10 anatomic specimens, each wrist undergoing 5 tests.

**Results:**

At 80° flexion mean elongation of the flexor carpi radialis (FCR) tendon slip was 0.96 ± 0.38 mm. Mean elongation of the dorsal intercarpal complex (DIC) was 3.75 ± 0.91 mm at 80° flexion. Relative elongation at the FCR tendon slip was 4.1% and 33.4% at the DIC. In 2 tests (4%), sutures loosened at the proximal side of the DIC after 30° flexion. Ruptures of the radiotriquetral ligament, pull-outs of the bone anchor or FCR tendon ruptures were not observed.

**Conclusion:**

This experiment suggests that following 3LT, the most elongation occurs at the DIC. Since instances of failure were noted at the proximal aspect of the DIC, if early active motion protocols are used, it is recommended to initiate wrist flexion with a limitation to 30° wrist flexion.

## Introduction

Injuries to the scapholunate joint are the most important cause of carpal instability.^1^ More recent, the concept of a stabilizing complex was introduced.^2^ This scapholunate complex consists of the scapholunate interosseous ligament (SLIL), the scaphotrapezio-trapezoid ligament, the radiolunate ligament, the radioscaphoid ligament, the dorsal intercarpal ligament and the flexor carpi radialis (FCR), extensor carpi radialis longus, and flexor carpi ulnaris muscles. Injuries of the scapholunate complex can lead to alterations of articular dynamics which can result in pain, decreased grip strength, and disability. Moreover, if left untreated, it may lead to osteoarthritis.^3^

Numerous surgical procedures for the treatment of instability have been proposed including direct repair, capsulodesis, tendon graft procedures, and arthroscopic repair. The aim of tendon grafts is to reduce flexion of the scaphoid and correcting dissociation between the scaphoid and lunate. The use of the FCR tendon was first described by Brunelli and has been modified by van den Abbeele and Garcia-Elias.^4-6^ Using the 3 ligament tenodesis (3LT) for management of scapholunate injuries is performed and advocated by multiple authors.^7,8^

The 3LT procedure is associated with an improvement of postoperative Disabilities of the Arm, Shoulder and Hand (DASH) scores, reduction of pain, and reduction of SL-gap.^9,10^ However, disadvantages are a decrease in wrist motion, especially flexion and grip strength.^6^

To improve the surgical procedure and the rehabilitation after surgery, a better understanding of the biomechanical properties after a 3LT procedure is necessary. For this reason, it would be valuable to understand which parts of the reconstruction are involved most in limiting flexion.

Second, according to the original case series of the 3LT technique, a 6-week postoperative immobilizing cast is indicated to protect the reconstruction.^6^ As a side effect, immobilization results in loss of mobility, stiffness, atrophy, and a longer rehabilitation period. If wrist movement does not appear to damage the surgical reconstruction, a faster start of the rehabilitation after surgery might be possible.

The objective of this anatomical specimen-based study is to evaluate the absolute and relative elongation of the FCR tendon slip and the dorsal intercarpal complex (DIC) during flexion. Secondary, this study aims to assess the reconstructions capacity to endure maximal flexion without failure. In the event of failure, we aim to objectify the nature of the failure.

## Methods

Ten specimens were disarticulated at the shoulder joint and embalmed according to the AnubiFiX method (AnubiFiX, Rotterdam, The Netherlands). This method is used to preserve tissue and joint flexibility.^11,12^ After disarticulation, all wrists were visually examined by a wrist surgeon for evidence of prior surgery and pathological changes. The 3LT was performed by the same surgeon according to the same surgical protocol.

### Surgical Technique

Following Berger and Bishops approach, an incision was made starting 1 cm ulnar of Lister’s tubercle.^13^ The underlying third and fourth extensor loge was opened. To open the overlaying capsule, an incision was made starting at the radial styloid, which moved ulnar until the center of the lunate fossa. From the lunate fossa, the incision moved in a distal direction following the fibers of the dorsal radiocarpal ligament until the insertion on the triquetrum. A second incision was made at the level of the scaphotrapezio-trapezoid joint and progressed in ulnar direction, splitting the DIC until its insertion on the triquetrum connecting the 2 incisions. The radially based capsular flap was elevated from the carpal bones. The SLIL was identified and transected entirely. At the volar side of the wrist, a slip was split from the FCR tendon and pulled dorsally through a drilled hole in the scaphoid. The FCR-tendon slip was fixed on the lunate using a bone anchor. The FCR-tendon slip was pulled through the radiotriquetral ligament (RTq), and sutured on itself. The radially based DIC flap was sutured to the surrounding tissue to restore the dorsal capsule.

### Measurement Technique

Each specimen was solidly fixed into a testing apparatus (Testometric Co, Rochdale, United Kingdom). A wire was sutured around the third metacarpal bone, guided through 2 fixed pulleys and attached to the vertically moving apparatus with a speed of 5.0 mm/s resulting in wrist flexion (Table 1). A digital goniometer was used for measuring flexion. Each specimen underwent 5 tests, ranging from neutral position to a maximum of 85°. Data extraction started when a preload of 0.5 N was reached.

**Table 1.** Elongation and Relative Elongation of the FCR Tendon Slip and the DIC During Wrist Flexion.

Wrist flexion (°)	FCR-tendon slip		DIC	
	Elongation (mm)	Δ elongation (%)	elongation (mm)	Δ elongation (%)
5	0.001 (±0.001)	0	0.15 (±0.17)	1.2
10	0.005 (±0.01)	0	0.29 (±0.22)	2.7
15	0.01 (±0.02)	0	0.29 (±0.30)	4.1
20	0.03 (±0.04)	0.2	0.58 (±0.28)	5.9
25	0.05 (±0.06)	0.4	0.93 (±0.46)	8.0
30	0.08 (±0.09)	0.6	1.29 (±0.52)	10.7
35	0.11 (±0.11)	0.8	1.63 (±0.58)	13.3
40	0.15 (±0.13)	1.1	1.98 (±0.66)	16.0
45	0.18 (±0.15)	1.3	2.29 (±0.72)	18.2
50	0.19 (±0.16)	1.5	2.56 (±0.77)	19.9
55	0.23 (±0.17)	1.8	2.88 (±0.84)	21.9
60	0.28 (±0.20)	2.2	3.18 (±0.91)	24.0
65	0.37 (±0.24)	2.9	3.42 (±1.03)	25.4
70	0.43 (±0.29)	3.3	3.67 (±1.14)	27.0
75	0.50 (±0.36)	3.3	3.58 (±1.17)	27.4
80	0.96 (±0.38)	4.1	3.75 (±0.91)	32.0
85	NA	NA	4.25 (±1.21)	33.4

*Note.* FCR = flexor carpi radialis; DIC = dorsal intercarpal complex.

**Figure 1.**
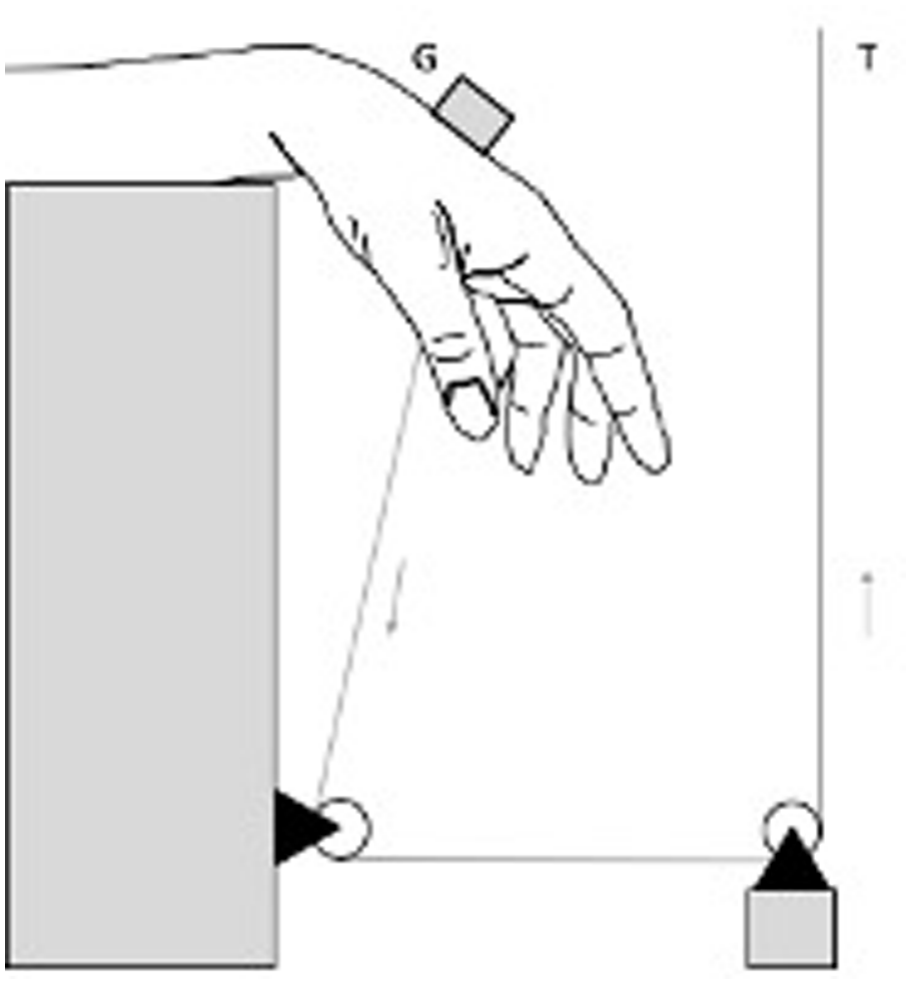
Schematic overview of testing apparatus. The universal testing machine (T) moved vertically, resulting in palmar flexion of the wrist. A digital goniometer (G) was used to quantify palmar flexion.

To assess elongation, a differential variable reluctance transducer (DVRT) (Microstrain, Burlington, Vermont) was placed over 2 intervals (Figure 1). To assess the elongation of the FCR-tendon slip, the DVRT was positioned in the FCR tendon where the slip left the scaphoid tunnel and at the bone anchor site. After testing elongation of the FCR-tendon slip, the DIC was sutured to restore the capsule. To quantify the elongation of the DIC, a second interval was used. The transducer was placed at Lister’s tubercle and at the site where the FCR-tendon slip was attached to the lunate. If the FCR-tendon slip or the DIC failed, the test was stopped.

Continuous variables were represented as mean and standard deviation. Failure was quantified through the count of observations and corresponding percentage of suture loosening, suture pull-outs, suture failures, bone anchor failures, FCR-tendon slip tears, and ligament tears.

## Results

No evidence for prior surgery or other significant pathology was observed. One of the anatomic specimens showed ossification at the SLIL during the surgical procedure. In all specimens the SLIL was intact. All wrists were tested 5 times resulting in 50 tests of the FCR-tendon slip and 50 of the DIC.

Mean elongation at the FCR-tendon slip was 0.96 ± 0.38 mm at 80° flexion. At 80° flexion mean DIC elongation was 3.75 ± 0.91 mm (Table 1). The mean starting lengths of the FCR-tendon slip and the DIC were 13.71 and 13.04 mm. Maximum relative elongation at the FCR tendon slip was 4.1%. Maximum relative elongation at the DIC was 33.4%.

No rupture of the RTq ligament, failure of the FCR tendon slip or pull out from the bone anchor was observed. In 2 wrists (2/50, 4%), suture loosening was observed at the proximal side of the DIC. Loosening occurred at 30° and at 55° flexion. No DIC ligament tears were seen.

## Discussion

Various studies explored the biomechanical properties of the SLIL. The biomechanical properties of the 3LT procedure are, despite its use, relatively unknown. Examination of the relation between wrist flexion and (relative) elongation and identification of failure during flexion might be valuable to improve the surgical treatment and faster recovery. This study found that during flexion more relative elongation is observed at the DIC compared with the FCR-tendon slip. Furthermore, loosening of sutures was observed at the proximal side of the DIC. No failure of the tendon graft, RTq ligament or bone anchor occurred.

Our study found that elongation at the DIC was 3.75 ± 0.91 mm during 80° flexion. This is in line with results from a prior biomechanical study, which reported an elongation of 3.9 ± 1.7 mm. after capsulodesis.^14^ Elongation at the FCR-tendon slip was 0.96 ± 0.38 mm, which was lower in comparison to the DIC. Similarly, more relative elongation was found in the DIC compared with the FCR-tendon slip. This difference might be caused by collinearity of the direction of the force and the interval measured, relative laxity of the DIC or the possibly that the DIC channels most forces during wrist flexion. Recent studies reported the existence of ligamentous attachment from the SLIL to the DIC and sectioning leaded to instability.^15,16^ This could suggest the DIC and its attachments have an important role in stabilization of the scapholunate interval after reconstruction.

The observation that loosening of sutures can occur at the proximal side of the DIC are consistent with a previous study concerning the biomechanics of capsulodesis, which found loosening of the proximal DIC sutures after 50° flexion.^14^ Care must be taken in suturing the proximal DIC.

Previous studies reported attention is needed during the procedure avoiding complications like ruptures of the RTq ligament, pull-outs of the bone anchor or FCR tendon ruptures.^6,17^ None of these complications were observed in our study. As relative elongation at the FCR tendon slip was only 4.1%, tensioning of the RTq might not be significant. However, this study only tested the 3LT with the same loading, more weight might alter elongation of the FCR tendon slip.

Nowadays, rehabilitation starts following an immobilization period of 6 to 8 weeks. If future research addresses early active motion, this study suggest initiating wrist flexion with a limitation to 30° wrist flexion as suture loosening occurred after 30° flexion.

This study has several limitations. First, all tests were performed on a limited number of wrists. Previous studies on SL pathology using anatomic specimen have involved 5 to 12 wrists.^18-20^ We believe testing 10 wrists, 5 times each, would be sufficient to make an adequate estimation of the flexion-elongation relation after 3LT. Second, like in all biomechanical studies, using postmortem human subjects concern the study conditions are not comparable with the in vivo reality. Especially in wrist surgery where scar tissue formation is thought to be an important aspect of the treatment, our model lacks this factor. Third, in this study we only transected the SLIL. Previous studies reported stabilization of the wrist is the result of primary and secondary stabilizers. To limit the effect of more than 1 variable, we only transected the SLIL. Fourth, due to the DVRT sensor limited accuracy in measuring elongation beyond 3.5 mm, repositioning of the sensor was needed at 45° during DIC tests. Presumably, this has influenced the elongation measurement of the DIC and contributed to a slight decrease in elongation around 45° flexion.

## Conclusions

The experiments performed suggest that most elongation is seen at the DIC after 3LT. Failure was only observed at the proximal side of the DIC where sutures loosened. These results might indicate the DIC has an important role in stabilizing the scapholunate interval after 3LT. The reconstruction seems to be stable enough to withstand flexion until 30°. Future studies should address earlier start of motion in a clinical setting and study the effect of shorter immobilization.
